# Recurrence and Frequency of Disturbance have Cumulative Effect on Methanotrophic Activity, Abundance, and Community Structure

**DOI:** 10.3389/fmicb.2015.01493

**Published:** 2016-01-05

**Authors:** Adrian Ho, Erik van den Brink, Andreas Reim, Sascha M. B. Krause, Paul L. E. Bodelier

**Affiliations:** ^1^Department of Microbial Ecology, Netherlands Institute of EcologyWageningen, Netherlands; ^2^Department of Biogeochemistry, Max Planck Institute for Terrestrial MicrobiologyMarburg, Germany; ^3^Department of Chemical Engineering, University of Washington, SeattleWA, USA

**Keywords:** recurring disturbance, *pmoA*, methane oxidation, functional traits, resilience

## Abstract

Alternate prolonged drought and heavy rainfall is predicted to intensify with global warming. Desiccation-rewetting events alter the soil quality and nutrient concentrations which drive microbial-mediated processes, including methane oxidation, a key biogeochemical process catalyzed by methanotrophic bacteria. Although aerobic methanotrophs showed remarkable resilience to a suite of physical disturbances induced as a single event, their resilience to recurring disturbances is less known. Here, using a rice field soil in a microcosm study, we determined whether recurrence and frequency of desiccation-rewetting impose an accumulating effect on the methanotrophic activity. The response of key aerobic methanotroph subgroups (type Ia, Ib, and II) were monitored using qPCR assays, and was supported by a t-RFLP analysis. The methanotrophic activity was resilient to recurring desiccation-rewetting, but increasing the frequency of the disturbance by twofold significantly decreased methane uptake rate. Both the qPCR and t-RFLP analyses were congruent, showing the dominance of type Ia/Ib methanotrophs prior to disturbance, and after disturbance, the recovering community was predominantly comprised of type Ia (*Methylobacter*) methanotrophs. Both type Ib and type II (*Methylosinus*/*Methylocystis*) methanotrophs were adversely affected by the disturbance, but type II methanotrophs showed recovery over time, indicating relatively higher resilience to the disturbance. This revealed distinct, yet unrecognized traits among the methanotroph community members. Our results show that recurring desiccation-rewetting before a recovery in community abundance had an accumulated effect, compromising methanotrophic activity. While methanotrophs may recover well following sporadic disturbances, their resilience may reach a ‘tipping point’ where activity no longer recovered if disturbance persists and increase in frequency.

## Introduction

Episodic desiccation-rewetting events are a phenomenon occurring in natural and anthropogenic-impacted environments, changing the soil quality (e.g., aggregate size distribution and soil organic matter) and nutrient concentrations (e.g., carbon and nitrogen), which in turn, drives microbial turnover in soils (Denef et al., 2001; Mikha et al., 2005). As such, recurring desiccation-rewetting may regulate soil microbial-mediated processes. Numerous studies determined the response of generalized processes (e.g., soil microbial respiration) and/or shifts in broad microbial phyla to desiccation-rewetting cycles or as a single event (Schimel et al., 1999; Denef et al., 2001; Fierer and Schimel, 2002; Fierer et al., 2003; Orwin and Wardle, 2004; Evans and Wallenstein, 2012). Although the frequency of the disturbance caused a significant decrease in soil respiration rate, the microbial community composition was rather insensitive to the desiccation-rewetting cycles (Fierer and Schimel, 2002; Fierer et al., 2003; Evans and Wallenstein, 2012). Because a microbial phylum may contain physiologically distinct members which respond differently to the disturbance, and that a shift in a broad microbial function catalyzed by members of multiple phyla may not be evident, the effects of disturbances may be more effectively captured by determining the response of a specialized microbial guild catalyzing a well-defined and specific process as has been shown for the ammonia-oxidizers (Placella and Firestone, 2013; Thion and Prosser, 2014) and methanotrophs (Levine et al., 2011).

Here, we used aerobic methanotrophs, representing a unique microbial guild that catalyzes a key biogeochemical process, and considered methane oxidation rate as the functional response variable. Aerobic methane oxidation is restricted to two phyla (*Proteobacteria* and *Verrucomicrobia*), with the proteobacterial methanotrophs forming the vast majority of the active population in many terrestrial ecosystems (Ho et al., 2013a). Verrucomicrobial methanotrophs are so far confined to low pH geothermal environments, typically below pH 5, albeit they have been detected in samples across a wide temperature range (Sharp et al., 2014). Canonical proteobacterial methanotrophs belong to the *Gammaproteobacteria* and *Alphaproteobacteria*, and is respectively represented by type Ia/Ib (family *Methylococcacceae*) and type II (families *Methylocystaceae* and *Beijerinkiaceae*) methanotrophs. The representative methanotrophs from these subgroups seemingly show different ecological characteristics and possess distinct traits associated to their life strategies (Ho et al., 2013a; Krause et al., 2014; Knief, 2015). Accordingly, the *pmoA* gene which encodes for a subunit of the particulate methane monooxygenase (pMMO) is conserved, and is congruent with the 16S rRNA gene phylogeny, making the use of the *pmoA* gene as means to detect and quantify methanotrophs suitable in culture-independent studies of complex environments (Kolb et al., 2003; Lüke and Frenzel, 2011).

Inducing disturbances as single events, aerobic methanotrophs showed remarkable resilience to prolonged drought (Collet et al., 2015), heat stress (Whittenbury et al., 1970; Ho and Frenzel, 2012), soil structural disruption (Kumaresan et al., 2011), and a disturbance-induced motility, monitoring re-colonization after a simulated die-off (Ho et al., 2011a; Pan et al., 2014). Although diversity decreased after disturbance, methanotrophic activity was not adversely compromised, and was even over compensated during recovery when compared to the un-disturbed community (Ho et al., 2011a; Kumaresan et al., 2011). These studies show that given time, methanotrophs are resilient to physical disturbances despite being a minority (<1.75%; Ho et al., 2011a; Lee et al., 2014, 2015) among the soil microorganisms. The resilience of natural methanotrophic communities can be attributed to their diverse traits, and hence, the adopted life strategies of the community members, enabling some methanotrophs to survive and persist, or even flourish under different environmental conditions and disturbances (Ho et al., 2013a).

Here, simulating a recurring disturbance, we induced a cyclic desiccation-rewetting event, and further increased the frequency of the disturbance by twofold (i.e., every 14 days to every 7 days) in a rice field soil. We aim to determine the response and recovery of aerobic methanotrophs to recurring desiccation-rewetting, and whether the frequency of the recurring disturbance imposed an accumulating effect on the soil nutrient concentrations and methanotrophic activity. We monitored the recovery of the methane uptake rates, as well as the response of the methanotrophic community abundance using group-specific quantitative PCR (qPCR) assays targeting the type Ia, Ib, and II methanotrophs. To follow shifts in community composition over time, we performed a *pmoA*-based t-RFLP analysis which was shown to have an adequate coverage of the methanotrophic diversity in this particular soil (Lüke et al., 2014).

## Materials and Methods

### Soil Microcosm and Experimental Setup

Rice field soil was sampled at the CRA Agricultural Research Council, Rice Research Unit (Vercelli, Italy) in May 2010. Soil parameters and rice agricultural practices in the sampling field have been described previously (Krüger et al., 2001). Soil, sampled at 0–20 cm depth, was air-dried at room temperature (∼22°C), crushed, sieved (2 mm), and stored covered in plastic containers prior to experimental set up. Approximately 60 microcosms were setup. Each microcosm contained 10 g air-dried soil filled in a sterile petri dish and saturated with autoclaved de-ionized water (0.45 ml per g dry soil). The microcosm was incubated in a gas tight jar at 25°C under 10%_v/v_ methane in air in the dark. Headspace atmosphere in the jar was replenished every 2–3 days to ensure constant air and methane availability. The microcosm was pre-incubated for 14 days to acclimatize to the incubation condition. Desiccation was induced by placing the microcosm under the laminar flow cabinet (Clean Air ES/FB, Telstar Life Science Solutions, Utrecht, the Netherlands) overnight (16 h) at room temperature (∼25°C) which caused >94% gravimetric water loss. Desiccation was induced fortnightly and weekly, designated as moderate and severe disturbances, respectively. After desiccation, water loss in the microcosm was replaced by adding the corresponding amount of autoclaved de-ionized water, and methane uptake rate was determined in triplicate. Water loss (∼2–3% gravimetric water content) in the un-disturbed microcosm was also replenished. After methane uptake measurement, the three microcosms (un-disturbed and disturbed microcosms, each) representing independent replicates were destructively sampled. The remaining microcosms were returned to the jar, and incubation was resumed under 10%_v/v_ methane in air in the dark. Depending on the disturbance, methane uptake was measured again after 7 or 14 days to determine the recovery of activity. Microcosm not exposed to desiccation served as a reference. The soil was homogenized before sampling, and stored in aliquots in the -20°C freezer till further analysis.

### Methane Uptake Rate

The methane uptake rate was determined as described before (Ho et al., 2011a; Ho and Frenzel, 2012). Briefly, the microcosm was removed from the jar, and placed in a flux chamber (volume: 172 ml). Methane (2–3 vol.%) was added into the headspace of the flux chamber. Methane uptake rate, determined from linear regression, was monitored over 4.5–5 h (4–5 sampling points). Methane concentration was determined using an Ultra GC gas chromatograph (Interscience, Breda, the Netherlands) equipped with a flame ionization detector (FID) and at Rt-Q-Bond (30 m, 0.32 mm, ID) capillary column. The oven temperature was 80°C, and helium was used as the carrier gas.

### Resilience Index, RL for Activity Measurement

The resilience index was derived by comparing methane uptake rates in the disturbed and un-disturbed microcosms using the following equation as proposed by Orwin and Wardle (2004):

$$RL\,\;at\,\;\,t_{x}=[2|D_{0}|/|D_{0}|+|D_{x}|]-1$$

where D_0_ and D_x_ refers to the difference in mean methane uptake rate between the disturbed and un-disturbed microcosms at time 0 (immediately after desiccation) and *x*, respectively (i.e., fortnightly and weekly for the moderately and severely disturbed microcosms, respectively). The RL value ranges from -1 to +1, with a value of +1 indicating full recovery (maximal resilience), and values <1 indicating slower rates of recovery. RL value of zero indicates no recovery at time *x* (since the end of disturbance; Orwin and Wardle, 2004). In this study, RL did not give a zero value. The resilience index was determined after every desiccation-rewetting cycle.

### Soil Nutrient Content

Nutrients (NO_x_, NH_4_^+^, and PO_4_^3-^) in the soil were determined using a SEAL QuA Atro SFA autoanalyzer (Beun-de Ronde B.V., Abcoude, the Netherlands) as described before (Ho et al., 2015). NO_x_ refers to the total of NO_2_^-^ and NO_3_^-^, and was below the detection limit.

### DNA Extraction

Total DNA was extracted from triplicate microcosms per treatment and time using the PowerSoil^®^ DNA Isolation kit (MOBIO, Uden, the Netherlands) as described in the manufacturer’s instruction.

### qPCR Assays

The qPCR assays, MBAC, MCOC, and TYPEII respectively targets the type Ia, type Ib, and type II methanotroph subgroups, were performed in duplicate per DNA extract. Additionally, the EUBAC assay was performed to enumerate the total 16S rRNA gene copies. The qPCR assays were performed with primers, primer concentration, and PCR profiles as given in **Table 1**. Each qPCR assay (total volume 20 μl) targeting the methanotrophs (MBAC, MCOC, and TYPEII assays) consisted of 10 μl 2X SensiFAST SYBR (BIOLINE, Alphen aan den Rijn, the Netherlands), 3.5 μl of forward and reverse primers each, 1 μl bovine serum albumin (5 mg ml^-1^; Invitrogen, Breda, the Netherlands), and 2 μl 100-fold diluted template DNA. Each EUBAC assay (total volume 15 μl) consisted of 7.5 μl 2X SensiFAST SYBR (BIOLINE), 0.75 μl of forward and reverse primers each, 1.5 μl bovine serum albumin (5 mg ml^-1^; Invitrogen), 3 μl 100-fold diluted template DNA, and 1.5 μl DNase- and RNase-free water. Plasmid DNA isolated from pure cultures was used for the calibration curve. Previously, in an initial qPCR run using the same soil, template DNA diluted by a 100-fold gave the optimal target yield (Ho et al., 2011a). The qPCR was performed using a Rotor-Gene Q real-time PCR cycler (Qiagen, Venlo, the Netherlands). Amplicon specificity was checked from the melt curve, and further confirmed by 1% gel electrophoresis which showed a single band of the correct size in the initial qPCR run.

**Table 1:** The primer sets and PCR thermal profile used in this experiment.

Primer set	Primer concentrations (forward/reverse)	PCR thermal profile^∗^	Data acquisition	qPCR assay	Reference
A189f/601r	700 nM/700 nM	94°C, 10 s; 54°C, 10 s; 72°C, 25 s	82°C, 8 s	MBAC	Kolb et al., 2003
A189f/468r	700 nM/700 nM	94°C, 10 s; 64°C, 10 s; 72°C, 25 s	85°C, 8 s	MCOC	Kolb et al., 2003
II223f/II646r	525 nM/525 nM	95°C, 10 s; 60°C, 10 s; 72°C, 25 s	87°C, 8 s	TYPEII	Kolb et al., 2003
EUB338f/EUB518r	250 nM/250 nM	95°C, 10 s; 53°C, 10 s; 72°C, 25 s	72°C, 5 s	EUBAC	Fierer et al., 2005


*Thermal profile showing temperature and time for denaturation, annealing, and elongation.

### pmoA-Based t-RFLP Analysis

The methodology for the t-RFLP, including the primer concentration, thermal profile, and subsequent comparative gene sequence analysis with a *pmoA* clone library derived from the same soil has been described in detail (Lüke et al., 2010; Ho et al., 2011a). Briefly, the *pmoA* gene was amplified from each DNA extract using the FAM-labeled A189f/mb661r primer pair prior to digestion with the restriction endonuclease *Msp*I. Next, the t-RFs were separated using the ABIPrism 310 (Applied Biosystems, Darmstadt, Germany), and comparison with an internal standard (MapMarker 1000; Bioventures, Murfreesboro, TN, USA) to determine the length of the t-RFs was performed with GeneScan 3.71 software (Applied Biosystems).

### Statistical Analysis

The t-RFLP profiles were standardized as described previously (Lüke et al., 2010, 2014). Briefly, the t-RFLP profiles were normalized to overall signal intensity and relative abundance was calculated using the R statistics software environment version (R Core Team, 2015). The correspondence analysis and heatmap were produced using the vegan version 2.3.0 (Oksanen et al., 2015) and gplots version 2.17.0 (Warnes et al., 2015) package, respectively. The level of significance (*p* < 0.01) between treatments and time was performed using ANOVA or *t*-test as implemented in SigmaPlot v12.5 (Systat Software, Inc., USA).

## Results and Discussion

### Response of Methanotrophic Activity to Recurring Desiccation-Rewetting

**FIGURE 1:**
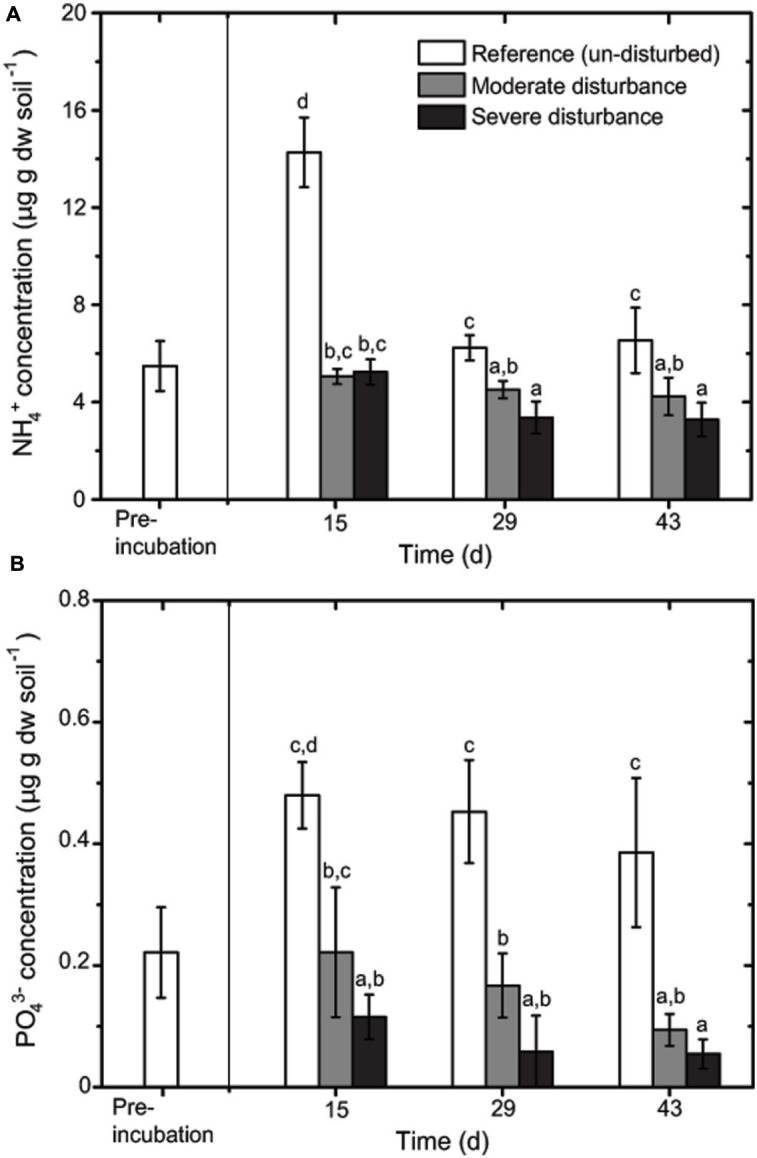
**Methane uptake rate during recovery from moderate **(A)** and severe **(B)** disturbances (mean ± SD; *n* = 3) determined after three (every 14 days) and six (every 7 days) consecutive cycles of desiccation-rewetting events, respectively.** Arrows indicate when desiccation was induced. Pre-incubation was performed for 14 days. The asterisk indicates the level of significance (*t*-test; *p* < 0.01) determined by comparing the methane uptake rates in the disturbed microcosms to the un-disturbed microcosms per time. In the moderately disturbed microcosms, and at cycles 1, 4, and 6 in the severely disturbed microcosms, methane uptake rate was determined immediately after the desiccation-rewetting event, but were not detected.

Methane uptake was detected in the un-disturbed microcosms, but ceased after desiccation-rewetting in the moderately disturbed microcosms (**Figure 1**). Although methane uptake was detected immediately after desiccation-rewetting in the severely disturbed microcosms, eventually reaching values which were significantly higher (cycle 3, *p* < 0.01; *t*-test) than in the un-disturbed microcosms during recovery, methane uptake became adversely affected after four consecutive cycles of desiccation-rewetting (**Figure 1**). At the final desiccation-rewetting event (cycle 6), methane uptake rate was significantly lower and activity did not recover to levels exhibited in the un-disturbed microcosm. The elevated methane uptake in response to severe disturbance in cycles two and three (**Figure 1**) is noteworthy, but remains to be fully elucidated. Previous studies showed the remarkable resilience of methanotrophic activity to distinct physical disturbances (e.g., disturbance-induced mortality; Ho et al., 2011a, heat stress; Whittenbury et al., 1970; Ho and Frenzel, 2012; prolonged drought; Collet et al., 2015, grinding; Kumaresan et al., 2011). These disturbances, however, were induced as a single event, and the response of methane uptake and the methanotrophic community composition were monitored during recovery. Given sufficient recovery period, methane uptake rate could be (over)compensated when compared to the un-disturbed incubation. Similarly, our results showed that recurring desiccation-rewetting events at moderate frequency appear to have no significant effect on the recovering methane uptake rate (**Figure 1**). However, increasing the frequency of the disturbance from every 14 to 7 days significantly compromise methanotrophic activity.

The resilience index, RL reflects the trend in methane uptake rate (**Supplementary Figure S1**), showing a decrease of the RL value after four cycles of desiccation-rewetting in the severely disturbed microcosm; in the third cycle, value was negative as anticipated because the recovering methane uptake rate was significantly higher than in the un-disturbed microcosm (Orwin and Wardle, 2004). The RL value decreased after the final desiccation-rewetting event in the moderately disturbed microcosms, suggesting that the methanotrophic activity was becoming less resilient with consecutive cycles. In contrast to previous work, this indicates a breaking point in the resilience of the methanotrophs despite of methane availability.

Given that site history can be an important determinant for contemporary microbial community composition and abundance, as well as functioning (Ho et al., 2011b; Evans and Wallenstein, 2012; Meisner et al., 2013; Thion and Prosser, 2014), it is not unreasonable to assume that samples sourced from other environments (e.g., deep lake sediments) may show less resilience. Here, we used a rice field soil which was repeatedly exposed to desiccation after drainage for rice harvest and subsequent flooding of the rice fields during the rice growing season, as per agriculture practice (Krüger et al., 2001). Hence, this soil has a legacy of periodic desiccation-rewetting stress which may have contributed to their resilience to our moderate disturbance regime. Increasing the frequency of the disturbance however, caused a breakdown in methanotrophic activity. Hence, methanotrophs indigenous to other environments without prior exposure to the disturbance may not be as resilient to recurring desiccation-rewetting.

### The Abiotic Environment

**FIGURE 2:**
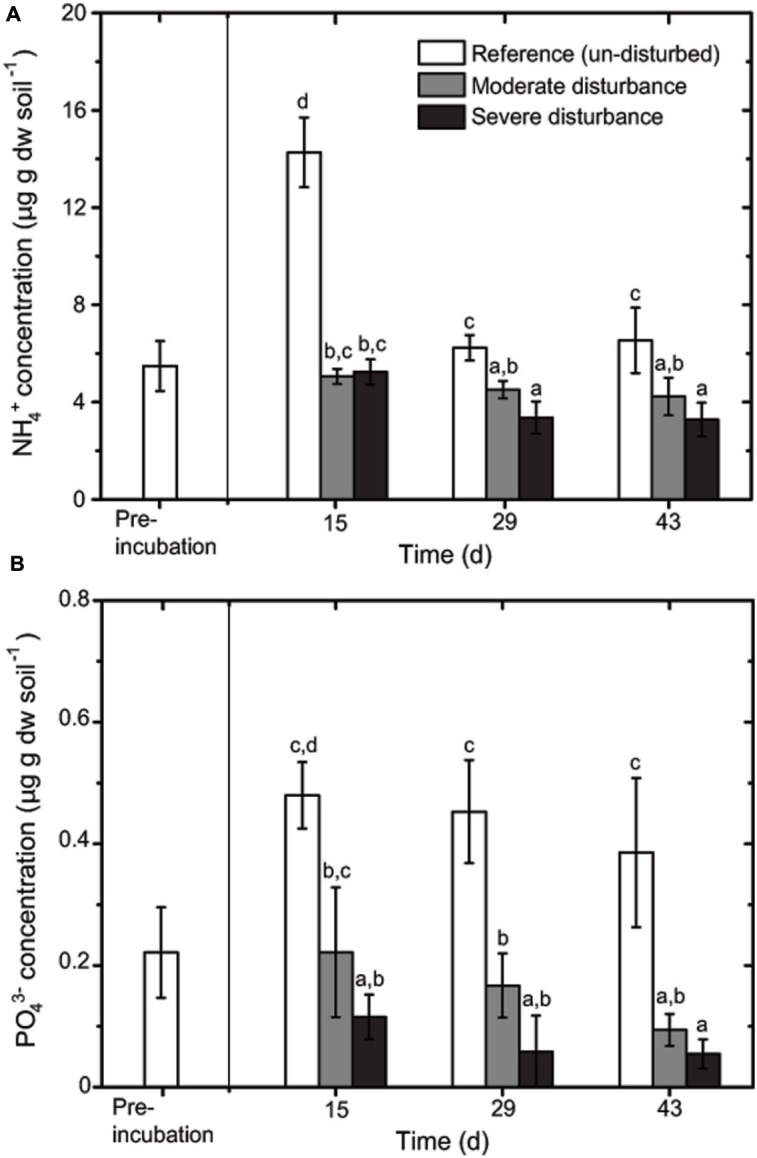
**Changes in NH_4_^**+**^**(A)** and PO_4_^3-^**(B)** concentrations during recovery from disturbances (mean ± SD; *n* = 3).** The different letters indicate statistical significance (ANOVA; *p* < 0.01) between treatments.

**FIGURE 3:**
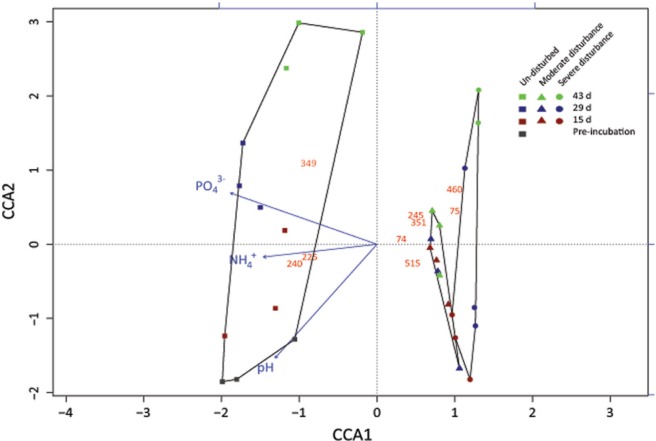
**Correspondence analysis showing the response of the methanotrophic community composition derived from the standardized *pmoA*-based t-RFLP data to the treatments (un-disturbed, moderate, and severe disturbances) and environmental variables (NH_4_^+^, PO_4_^3-^, pH, and methane oxidation rate).** The t-RFLP analysis was performed for each DNA extract (*n* = 3) per time; all replicates are given in **Supplementary Figure S2**. Red, blue, and green symbols indicate 15, 29, and 43 days, respectively. This corresponds to cycles 1, 2, and 3 in the moderately disturbed microcosms, and cycles 2, 4, and 6 in the severely disturbed microcosms. Gray symbol indicate the initial sampling point after pre-incubation, before disturbance was induced. The red numbers indicate the t-RFs; unidentifiable t-RFs are not shown. Methanotrophs affiliated to the t-RFs are given in **Supplementary Figure S2**.

**FIGURE 4:**
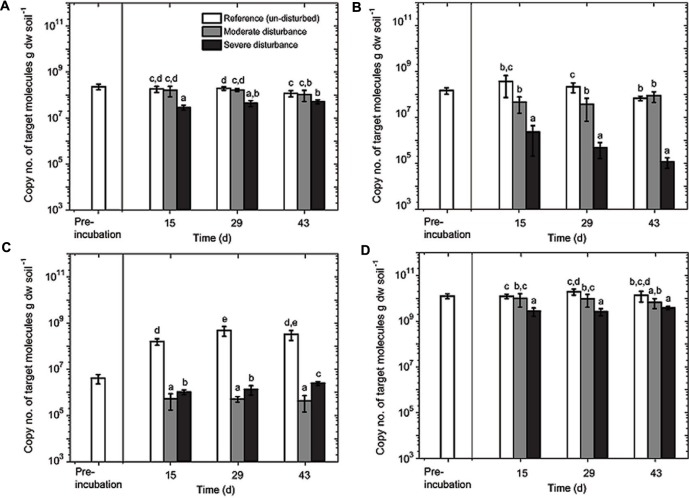
**Quantitative polymerase chain reaction (qPCR) analysis of MBAC **(A)**, MCOC **(B)**, TYPE II **(C)**, and EUBAC **(D)** assays.** The assays were performed in duplicate from triplicate DNA extracts (mean ± SD; *n* = 6) per time and treatment (un-disturbed, moderate, and severe disturbances). Pre-incubation was performed for 14 days. Sampling days 15, 29, and 43 correspond to cycles 1, 2, and 3 in the moderately disturbed microcosms, and cycles 2, 4, and 6 in the severely disturbed microcosms. All data points (8, 15, 22, 29, 36, and 43 days corresponding to cycles 1 through 6) for the severely disturbed microcosms are given in **Supplementary Figure S3**. The different letters indicate statistical significance (ANOVA; *p* < 0.01) between treatments during the recovery from disturbances.

Desiccation and subsequent rewetting may have caused a carbon/nitrogen flush, mobilizing nutrients from the soil and/or increasing soil nutrients as a result of cell lysis (Kieft et al., 1987; Mikha et al., 2005). These nutrients can be rapidly consumed by the recovering microorganisms upon rewetting as indicated in our study (**Figure 2**). NH_4_^+^ and PO_4_^3-^ concentrations were negatively affected by the disturbance regime; NH_4_^+^ and PO_4_^3-^ concentrations was significantly lower in both the disturbed microcosms (**Figures 2** and **3**), but changes in the total nutrient concentrations were not pronounced over time suggesting that the nutrient pool available remained relatively constant throughout the desiccation-rewetting cycles (**Figure 2**). However, we cannot completely exclude that nutrient limitation may be a factor restricting the methanotroph population size during the recovery from disturbance. Nevertheless, considering that NH_4_^+^ and PO_4_^3-^ concentrations were comparable in both the moderately and severely disturbed microcosms, while the community abundance was significantly affected (i.e., a decrease and increase in type Ib and type Ia/II *pmoA* gene abundance, respectively), suggest that the shift in the methanotroph subgroups were unlikely constrained by nutrient availability (**Figures 2** and **4**). Although the pH was significantly affected by the disturbance (*p* < 0.01; **Figure 3**) as revealed by the correspondence analysis, the values shifted only within a narrow range of 0.2 and 0.1 units in the disturbed and un-disturbed microcosms, respectively. Hence, we do not anticipate major effects of pH shift on the methanotrophic activity.

### Response of Methanotrophic Abundance and Composition to Recurring Desiccation-Rewetting

The aerobic methanotrophic composition in this rice field soil is well-characterized, comprising of methanotrophs belonging to the families *Methylococcaceae* (type Ia and Ib) and *Methylocystaceae* (type II), with the type II methanotrophs (*Methylocystis–Methylosinus*) being numerically dominant (Kolb et al., 2003; Ho et al., 2011a; Lüke et al., 2014). However, considering the *pmoA* gene transcript as a proxy for potential methanotrophic activity, methanotrophs belong to the subgroup type Ia (e.g., *Methylobacter*) appears to form the predominantly active population in rice field soils (Reim et al., 2012; Ho et al., 2013b; Ma et al., 2013; Collet et al., 2015). Hence, we monitored the *pmoA* gene abundance of these subgroups (type Ia, Ib, and II methanotrophs), as well as the total 16S rRNA gene during the recovery from disturbances. The lower detection limit of the qPCR assays was 10^3^–10^4^ copies of target molecule g dry weight soil^-1^, depending on the assay; in all samples, the *pmoA* and 16S rRNA gene copies were above the detection limit (**Figure 4**). The disturbance exerted a differential response among the methanotroph subgroups (**Figure 4**). The *pmoA* gene abundances of type Ia and Ib methanotrophs remained relatively stable after moderate disturbance; type Ia *pmoA* gene abundance being the least responsive to the induced disturbances. In contrast, type II *pmoA* copies were reduced by approximately two orders of magnitude after both moderate and severe disturbances. In the un-disturbed microcosms, however, type II *pmoA* gene abundance initially increased (<15 days; **Figure 4**) before reaching a stable abundance. Although not appreciably affected by the moderate disturbance, *pmoA* gene belonging to type Ib methanotrophs significantly decreased by 2–3 orders of magnitude after severe disturbance. The decrease was not statistically significant over time, but the trend indicates that type Ib methanotrophs were negatively affected with increasing desiccation-rewetting frequency (**Figure 4**). Hence, results showed the differential response in the methanotroph subgroups to recurring desiccation-rewetting (frequency), indicating the inherently different degrees of resilience to the disturbance.

Although changes in community composition may not indicate a change in activity, it can be significantly related to the shift in population size (Ho et al., 2011b). Shifts in community composition following disturbance was determined by a correspondence analysis derived from the t-RFLP data using environmental variables (NH_4_^+^, PO_4_^3-^, and pH) as constraints, of which NH_4_^+^, PO_4_^3-^, and pH showed statistically significant correlation (*p* = 0.001, 0.001, and 0.002, respectively) together explaining 34.64% of total variance (**Figure 3**). Consistent with the qPCR analysis, the ordination revealed that the community composition in the un-disturbed and disturbed microcosms diverged. Besides the t-RFs identifiable from comparison to a *pmoA* clone library of the same soil (Lüke et al., 2010), with the exception of t-RFs 223 and 144, other t-RFs (t-RFs 227, 268, 277, 243, 289, and 338) which could not be associated to known methanotrophs represented only a minor fraction of the total community (**Supplementary Figure S2**). The relative abundance of t-RF indicative for type II methanotrophs (t-RF 245) increased in the un-disturbed microcosm, but the increase was more pronounced after severe disturbance, while type Ia/Ib-related t-RFs (t-RF 515 and 74) were dominant in both the disturbed microcosms (**Figure 3** and **Supplementary Figure S2**). Given that the t-RFLP analysis detects the shift in the relative abundance of the community composition, the increase in type II-related t-RF (t-RF 245) in the severely disturbed microcosm may be caused by the numerical decrease in the type Ib subgroup (∼10^6^ to ∼10^5^ *pmoA* gene copies; **Figure 4**), while the type Ia subgroup remained relatively constant. Nevertheless, type II *pmoA* gene abundance was significantly, if not appreciably higher after six consecutive desiccation-rewetting events (**Figure 4** and **Supplementary Figure S3**). In particular, t-RF 515 indicative for *Methylobacter* (type Ia methanotroph) responded positively to increasing desiccation-rewetting intensity, whereas t-RF 74 indicative for M*ethylocaldum*/*Methylococcus* (type Ib methanotrophs) formed the majority during the recovery from moderate disturbance, but relative abundance was reduced after severe disturbance (**Supplementary Figure S2**). The compositional dynamic indicates a transition in the trajectory of community composition and abundance as the frequency of the desiccation-rewetting event increased. Overall, both the qPCR and t-RFLP analyses were congruent, showing that the recovering community was predominantly comprised of type Ia (*Methylobacter*), and that although type II (*Methylosinus*/*Methylocystis*) methanotrophs were initially adversely affected by desiccation-rewetting, population increased over time.

### Methanotroph Community Ecology

Recurring desiccation-rewetting may favor some community members capable of adjusting to rapid changes in the water potential, and capitalize on the sudden nutrient availability (i.e., strong competitors/ruderals represented by type Ia/Ib genera; Ho et al., 2013a). However, with further consecutive desiccation-rewetting cycles for prolonged periods, other methanotrophs (i.e., stress tolerators represented by type II genera; Ho et al., 2013a) were hypothesized to prevail. Notably, the type Ib methanotrophs (*Methylococcus*/*Methylocaldum*) were adversely affected by the frequency of the disturbance. Some *Methylococcus*/*Methylocaldum* genotypes are known to be thermotolerant, if not thermophilic, and were isolated from relatively harsh environments (e.g., hot springs; Bodrossy et al., 1999; Trotsenko et al., 2009). Here, although type Ib methanotrophs showed resilience to recurring desiccation-rewetting, upon increasing the frequency of the disturbance, they become susceptible, suggesting that rice field soil *Methylococcus*/*Methylocaldum*-related methanotrophs are not as well-adapted to rapidly changing environmental conditions as other community members. On the contrary, *Methylobacter*-related type Ia methanotrophs showed remarkable resilience to recurring desiccation-rewetting and remained relatively unperturbed by the frequency of the disturbance, although they form relatively less resistant resting cells (Whittenbury et al., 1970). Hence, some *Methylobacter* may possess yet unrecognized traits pertinent under conditions of fluctuating osmolarity caused by desiccation and rewetting.

Unexpectedly, type II methanotrophs were decimated even after moderate disturbance and numbers had not recovered (**Figure 4**). Despite being able to form heat- and desiccation-resistant exospores and/or lipid cysts (Whittenbury et al., 1970), type II methanotrophs seemingly do not function well under desiccation-rewetting. Moreover, Murase and Frenzel (2008) showed that protozoa do not preferentially graze on type II methanotrophs (*Methylocystis* sp.) in a rice field soil, potentially eliminating the likelihood of protozoa grazing in reducing type II methanotroph abundance. However, regardless of the increased disturbance frequency, the type II methanotrophs were no longer adversely affected after the initial (cycle 1) desiccation-rewetting event, and showed a relatively stable community with a significant increase in population size over time. This was consistent with a previous study showing recovery (relative abundance) of type II methanotrophs following two alternating dry/wet cycles over 80 days (Ma et al., 2013). Possibly, the increase in type II population was represented by the (emerging) seed bank population. More generally, it is not unreasonable to assume that the type II, as well as type Ia methanotrophs may have re-colonized space made available from the die-off of other microorganisms in the severely disturbed microcosms as shown before (**Figure 4**; Ho et al., 2011a; Pan et al., 2014). Therefore, our disturbance regime did not only determine the resilience of the methanotrophic activity to recurring desiccation-rewetting (frequency), but also revealed the ecological characteristics of different community members.

## Conclusion

Taken together, this study along with previous work (Whittenbury et al., 1970; Ho et al., 2011a; Kumaresan et al., 2011; Ho and Frenzel, 2012; Collet et al., 2015) suggest that while methanotrophs may recover well following sporadic disturbances, their resilience may reach a ‘tipping point’ if disturbance persists and increased in frequency. However, our conclusion comes with a caveat: in designing the experiment to test the resilience of the methanotrophs and the accumulating effect of recurring disturbance, we induced short-term desiccation-rewetting cycles at regular intervals in a microcosm study which may not reflect field conditions, albeit a natural community of aerobic methanotrophs was used. Moreover, to achieve a standardized incubation condition, methane was continuous supplied in excess during the recovery from the disturbance. Since methanogenesis is likely affected by desiccation too, methane availability would have been disrupted under field conditions. Nevertheless, regardless of the duration and interval of the disturbance, as well as the excess supply of methane, our results show that recurring disturbances before a full recovery in the community abundance have a cumulative effect which compromises methanotrophic activity.

## Author Contributions

AH and PB conceived and designed the study. AH, AR, and EvdB performed the work, and acquired and analyzed the data. AH wrote the manuscript. EvdB, AR, SK, and PB critically revised and approved the manuscript. All authors are accountable for all aspects of the work.

## Conflict of Interest Statement

The authors declare that the research was conducted in the absence of any commercial or financial relationships that could be construed as a potential conflict of interest.
