# Supplementary figures and images for: Recurrence and Frequency of Disturbance have Cumulative Effect on Methanotrophic Activity, Abundance, and Community Structure
Source: Front Microbiol. 2016 Jan 5;6:1493. doi: 10.3389/fmicb.2015.01493 (PMC4700171; doi:10.3389/fmicb.2015.01493)

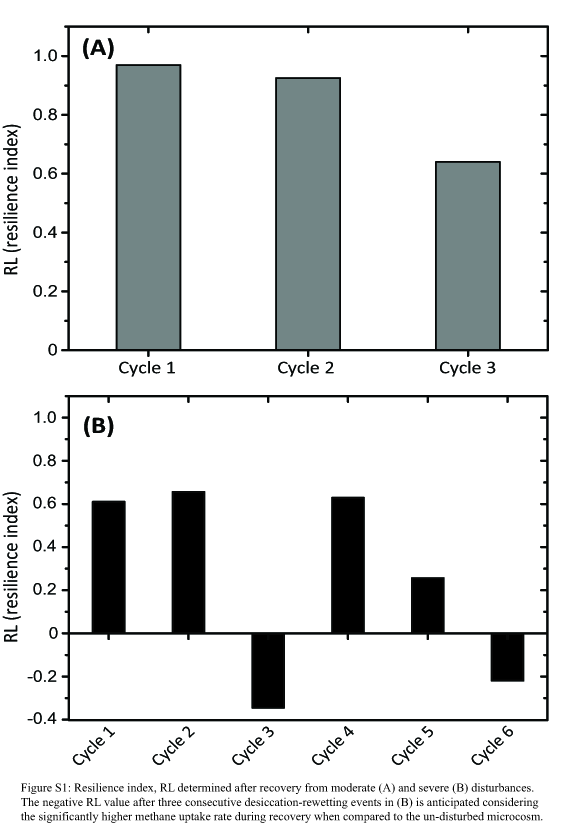

Supplement: Supplementary file 1 [file Image_1.TIF]

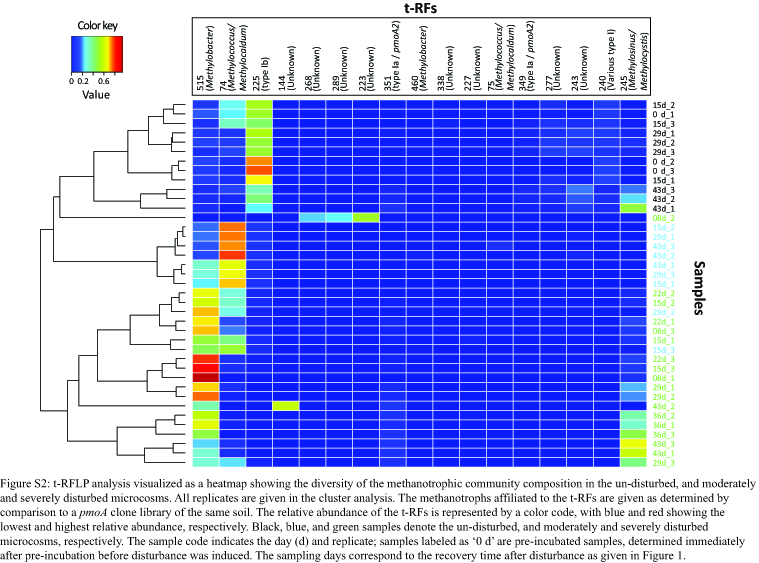

Supplement: Supplementary file 2 [file Image_2.TIF]

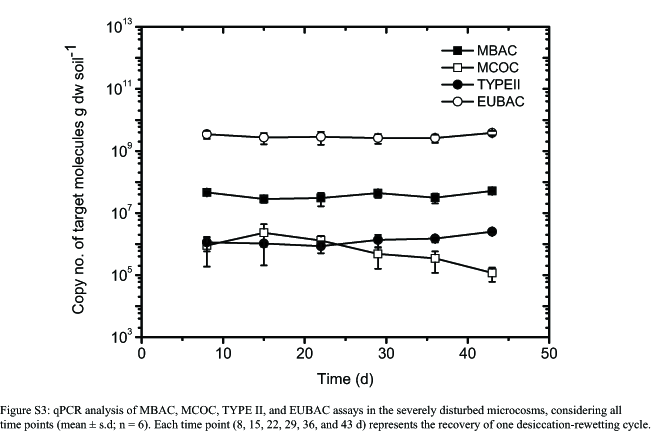

Supplement: Supplementary file 3 [file Image_3.TIF]
